# Pigtail Suprapubic Catheter Placement in a Patient With Acute Urinary Retention and Abdominal Mesh

**DOI:** 10.7759/cureus.107175

**Published:** 2026-04-16

**Authors:** Clara Keum, Zamran Masih, James Espinosa, Alan Lucerna

**Affiliations:** 1 Emergency Medicine, Jefferson Health New Jersey, Stratford, USA

**Keywords:** emergency department management of urinary retention, pigtail catheter use in urinary retention, suprapubic catheter placement, treatment of acute urinary retention, urinary retention

## Abstract

Acute urinary retention (AUR) is a urological emergency that requires immediate bladder decompression. When urethral catheterization is not feasible, suprapubic catheterization can be performed as an alternative. An 86-year-old male patient with a history of benign prostatic hyperplasia and prior hernia repair with surgical mesh presented to the emergency department (ED) with AUR for 16 hours. Due to multiple false passages, a completely blind-ended bladder neck, and a 6 mm urethral stone, urethral catheterization was unsuccessful, prompting an attempt at suprapubic catheterization. However, suprapubic catheter placement was also unsuccessful due to the presence of surgical mesh from the previous hernia repair. Instead, an 8.5 Fr pigtail catheter was successfully placed using ultrasound guidance and the Seldinger technique, draining over 1 L of urine from the patient’s bladder. The use of a smaller-diameter pigtail catheter offers multiple benefits when standard suprapubic catheter placement is not feasible due to anatomical or structural difficulties. It allows for urgent and effective bladder decompression, minimal training is required, and pigtail catheters are readily accessible. However, because smaller-diameter catheters are used, they may not effectively drain blood clots. Despite this limitation, their availability and effectiveness make them a viable alternative in the ED.

## Introduction

Acute urinary retention (AUR) is a urological emergency requiring prompt bladder decompression to relieve patient discomfort and prevent complications such as infection and renal dysfunction [1-3]. It is most commonly caused by bladder outlet obstruction, particularly in older males with benign prostatic hyperplasia, though a wide range of obstructive, neurologic, infectious, and pharmacologic etiologies exist [1,3].

When urethral catheterization is unsuccessful or contraindicated, suprapubic catheterization provides an effective alternative for bladder drainage. This procedure is commonly performed using a percutaneous Seldinger technique and may be facilitated by ultrasound guidance, particularly in patients with complex anatomy [4-6].

However, in patients with structural or surgical challenges, such as prior abdominal mesh placement, fibrosis, or altered anatomy, standard suprapubic catheter placement may be technically difficult or unsuccessful. In such scenarios, adaptation of existing wire-guided access techniques and alternative catheter devices may provide a practical solution [5].

We present a case of successful ultrasound-guided placement of an 8.5 Fr pigtail catheter for bladder decompression in a patient with AUR and prior abdominal mesh. This case highlights a readily available and effective alternative when standard suprapubic catheterization techniques fail and underscores the importance of procedural adaptability in managing complex urologic presentations in the emergency setting.

## Case presentation

An 86-year-old male patient with a history of coronary artery disease (CAD), benign prostatic hyperplasia (BPH), prior hernia repair with surgical mesh placement, multiple UroLift procedures, prostate reduction procedures, and resection of a bladder neck stricture presented to the ED with AUR. On initial evaluation, the patient reported a gradual decline in urine output over several weeks despite increased urinary urgency, culminating in an inability to void for the preceding 16 hours. He described significant suprapubic discomfort radiating to the lower back, as well as worsening left-sided testicular pain over the prior month

On examination, the patient appeared uncomfortable. Vital signs were notable for a blood pressure of 170/83 mmHg, with otherwise normal heart rate, respiratory rate, and temperature. Physical examination revealed suprapubic tenderness and mild left scrotal swelling without penile swelling, drainage, rash, erythema, or warmth. The remainder of the examination was unremarkable.

Initial laboratory evaluation demonstrated leukocytosis with a white blood cell count of 12.3 × 10³/mm³. Renal function was preserved, with a creatinine of 0.9 mg/dL and blood urea nitrogen of 15 mg/dL. Additional laboratory and urinalysis findings are summarized in Table 1.

**Table 1:** Laboratory and urinalysis findings BUN: blood urea nitrogen; PT: prothrombin time; PTT: partial thromboplastin time; INR: international normalized ratio; AST: aspartate aminotransferase; ALT: alanine aminotransferase; K/uL: thousands per microliter; g/dL: grams per deciliter; mEq/L: milliequivalents per liter; mg/dL: milligrams per deciliter; mmol/L: millimoles per liter; IU/L: international units per liter; mm³: cubic millimeter; cells/HPF: cells per high-power field

Laboratory results	Result	Normal range	Units
White blood cell count	12,300	4.0-11.0	mm^3^
Hemoglobin	14.0	10.6-15.6	g/dL
Platelet count	231	150-400	K/uL
Sodium	139	135-154	mEq/L
Potassium	4.4	3.5-5	mEq/L
BUN	15.0	5 to 20	mg/dL
Creatinine	0.9	0.6-1.2	mg/dL
Glucose	95.0	70-100	mg/dL
Calcium	8.8	8.5-10.5	mg/dL
Chloride	101.0	95-105	mEq/L
Bicarbonate	28.0	23-29	mEq/L
Magnesium	1.8	1.7-2.2	mg/dL
lactate	1.1	0.5-2.2	mmol/L
PT	11.0	11-13.5	sec
PTT	33.0	25-35	sec
INR	1.0	0.8-1.1	INR ratio
Urine color	Yellow	Yellow	NA
Urine clarity	Hazy	Clear	NA
Urine specific gravity	1.020	1.005-1.030	NA
Urine pH	7.4	5 to 7.5	NA
Urine glucose	Negative	Negative	NA
Urine protein	3 plus	Negative	NA
Urine bilirubin	Negative	Negative	NA
Urine urobilinogen	Negative	Negative	NA
Urine ketones	Negative	Negative	NA
Urine blood	3 plus	Negative	NA
Urine white cells	11 to 20	0-5/HPF	cells/HPF
Urine red cells	12 to 20	0-5/HPF	cells/HPF
Urine nitrite	Negative	Negative	NA
Urine leukocyte esterase	2 plus	Negative	NA
urine culture (after catheter placement)	<10.000 mixed Gram-positive, 10,000 to 40,000* Pseudomonas aeruginosa*	Negative	organisms/ml

**Figure 1:**
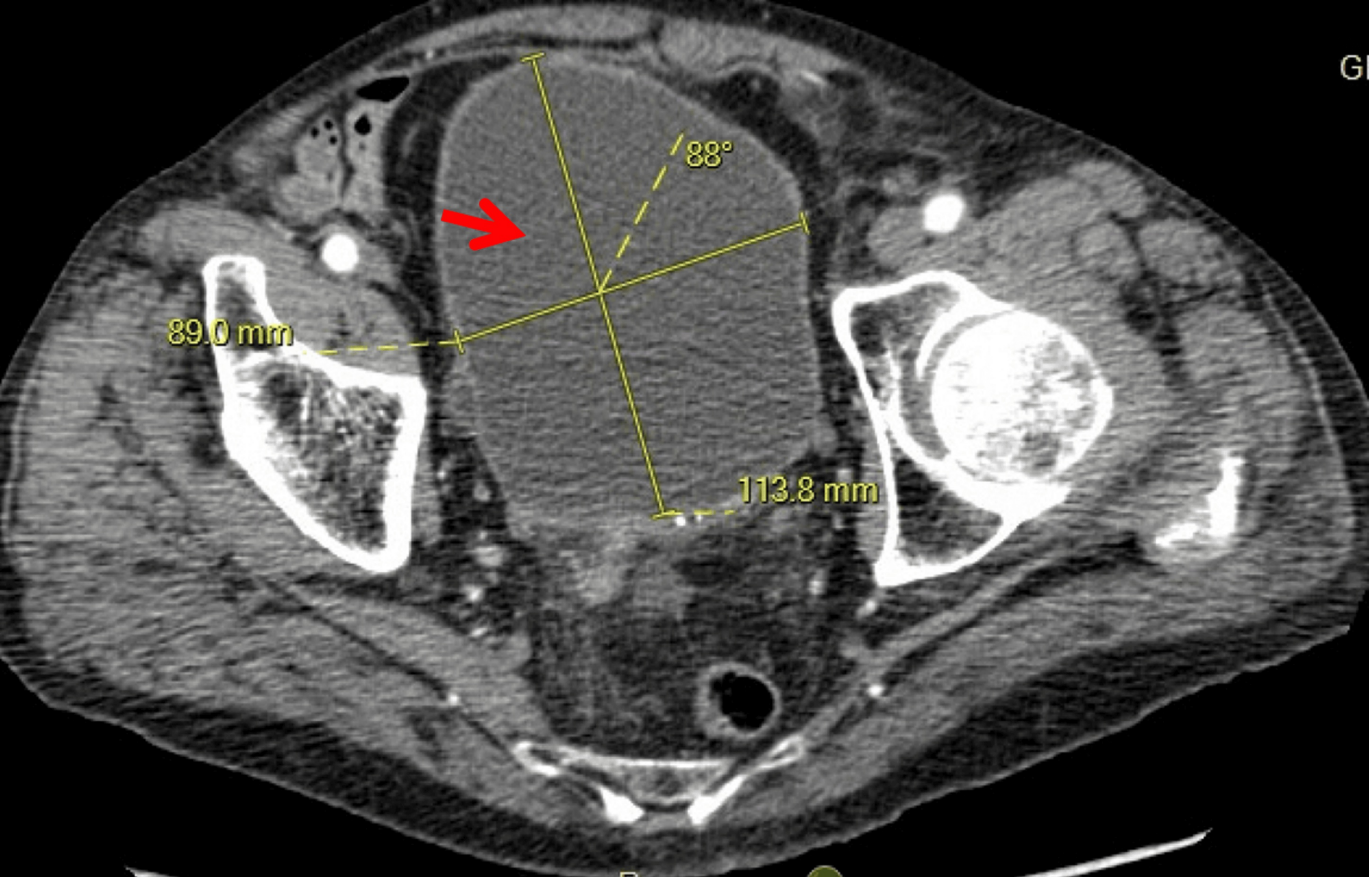
CT abdomen and pelvis, axial view. Markedly distended urinary bladder (arrow), consistent with acute urinary retention

**Figure 2:**
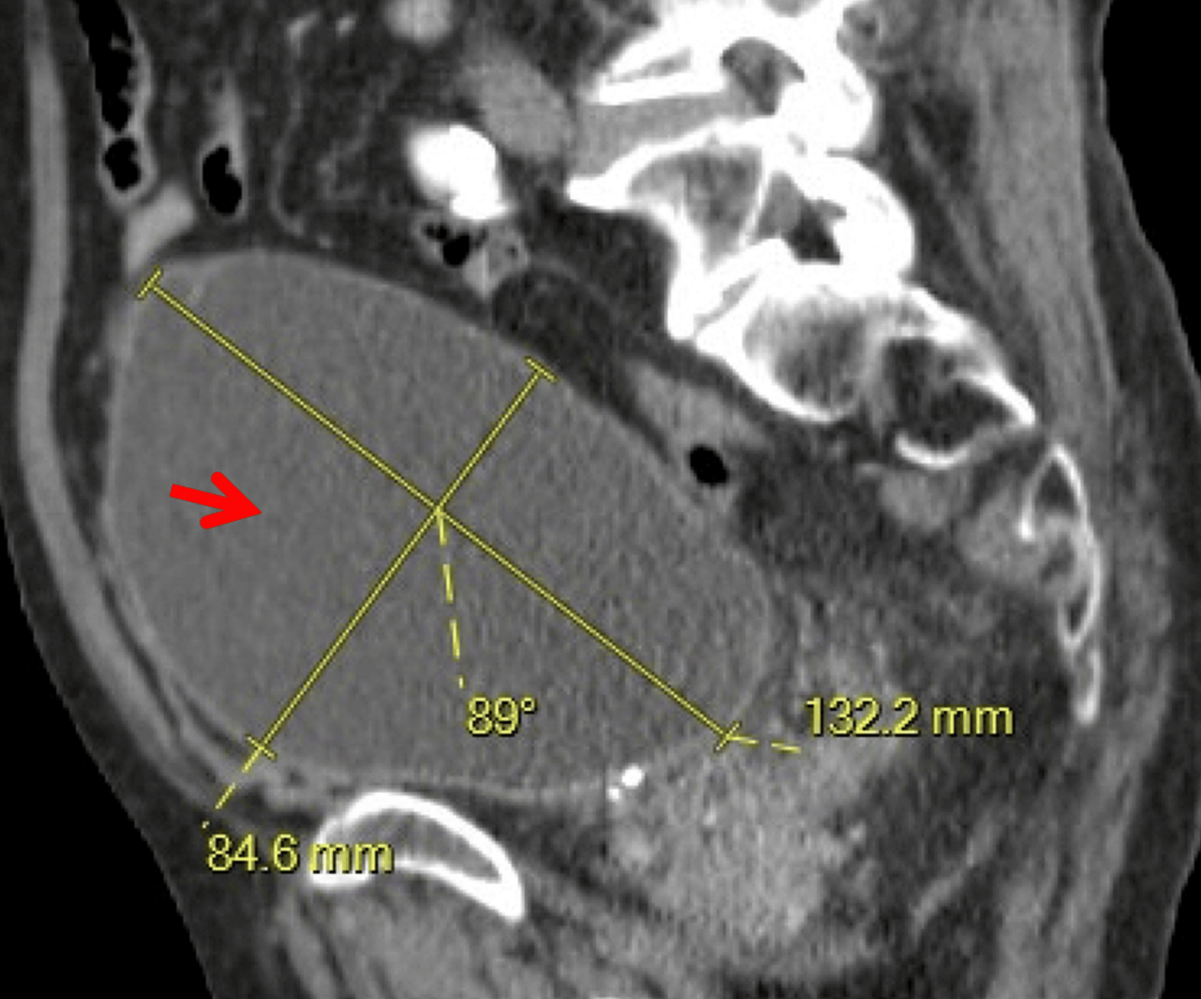
CT abdomen and pelvis, sagittal view. Severely distended urinary bladder (arrow) extending superiorly within the pelvis, consistent with acute urinary retention

**Figure 3:**
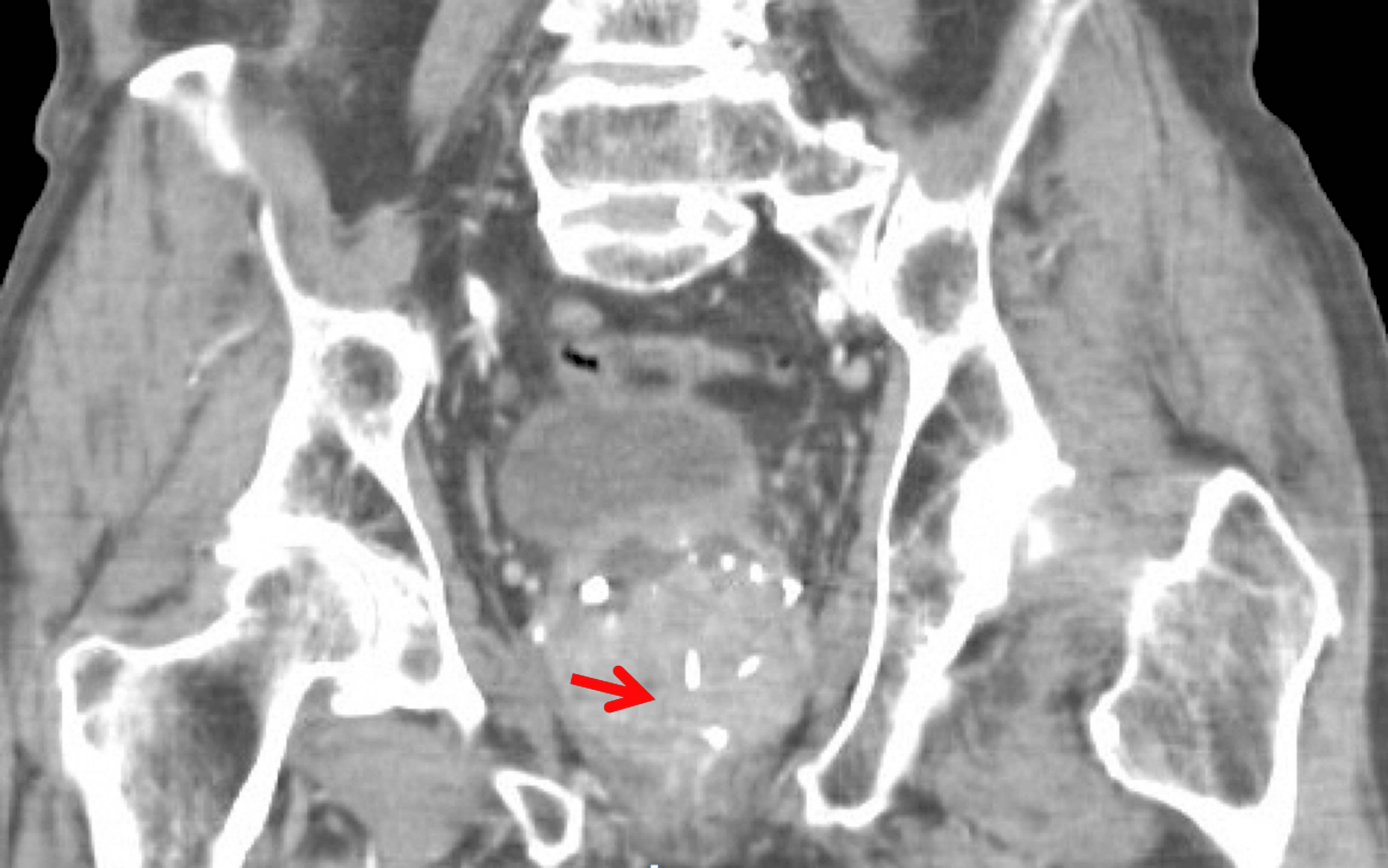
CT abdomen and pelvis, coronal view. Enlarged prostate gland (arrow) contributing to bladder outlet obstruction

**Figure 4:**
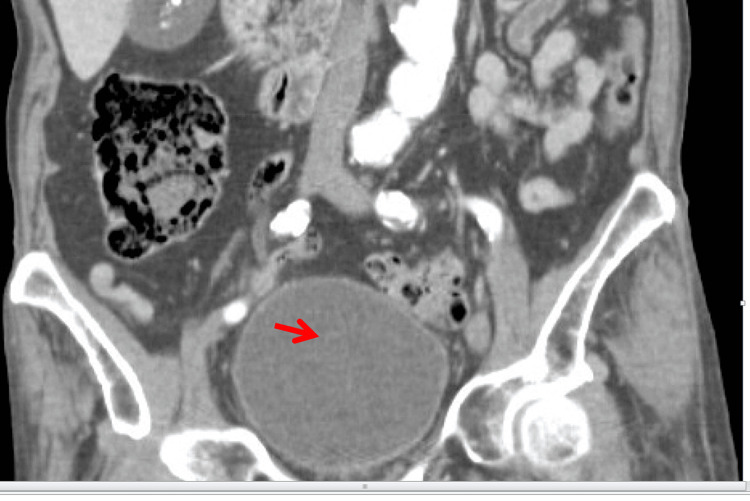
CT abdomen and pelvis, coronal view. Distended urinary bladder (arrow), consistent with bladder outlet obstruction

**Figure 5:**
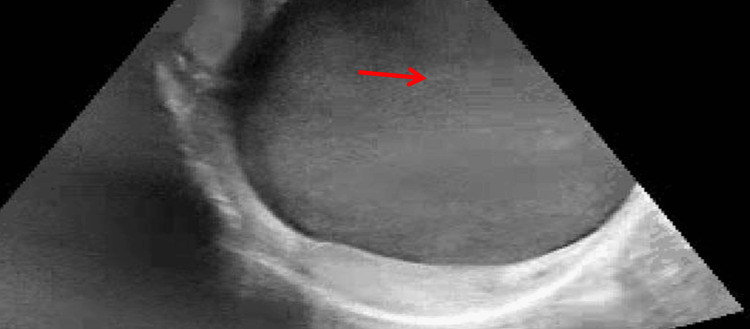
Scrotal ultrasound demonstrating left-sided hematocele Ultrasound of the left scrotum demonstrating an enlarged, complex fluid collection (arrow), consistent with a hematocele

Given concern for obstructive pathology, further diagnostic evaluation was pursued. A bladder scan revealed 475 mL of retained urine. Contrast-enhanced computed tomography (CT) of the abdomen and pelvis demonstrated a markedly enlarged prostate gland with associated bladder distension and wall thickening, consistent with bladder outlet obstruction. Additionally, a complex fluid collection was identified within the left scrotum, consistent with a hematocele, without evidence of hernia (Figures 1-4). Testicular ultrasound confirmed an enlarged, complex fluid collection within the left scrotum, further supporting the diagnosis of hematocele (Figure 5).

Initial attempts at bladder decompression were unsuccessful. Despite multiple attempts, urology residents were unable to place a suprapubic catheter due to the presence of prior abdominal mesh. Bedside cystoscopy further revealed multiple false passages, a completely blind-ended bladder neck, and a 6 mm urethral stone, making urethral catheterization with a Foley catheter technically challenging.

**Figure 6:**
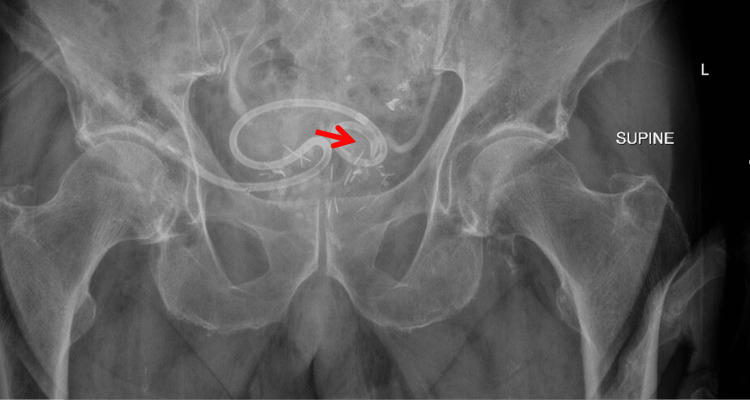
Pelvic X-ray following suprapubic catheter placement Pelvic radiograph demonstrating appropriate positioning of the pigtail catheter within the bladder, with visualization of the characteristic coiled tip (arrow)

Given these anatomic and procedural challenges, an alternative approach to bladder decompression was pursued. Under ultrasound guidance, an 8.5 Fr locking drainage pigtail catheter was placed suprapubically using a wire and dilator from a central line kit via the Seldinger technique. The catheter was secured to the abdominal wall with 2-0 silk sutures. Successful placement was confirmed by immediate urine return and pelvic radiography demonstrating appropriate positioning of the catheter with visualization of the characteristic coiled tip within the bladder (Figure 6).

Upon catheter placement, approximately 700 mL of urine was drained, with a total recorded output exceeding 1 L per chart review. Postcatheter placement urinalysis revealed hematuria and bacteriuria (Table 1). The patient was hospitalized with urology and infectious disease consultations. Upon confirmation of *Pseudomonas *cystitis, sensitive to cefepime, the patient was treated with cefepime per infectious disease recommendations during hospitalization. He was subsequently discharged with oral ciprofloxacin, hyoscyamine, and phenazopyridine, with outpatient urology follow-up. No additional outpatient follow-up data were available at the time of this report.

## Discussion

AUR is a urologic emergency defined by the inability to void despite a distended bladder and requires prompt recognition and intervention. The etiology of AUR is multifactorial and includes obstructive, neurologic, infectious, inflammatory, and pharmacologic causes. In male patients, bladder outlet obstruction, most commonly due to benign prostatic hyperplasia, is the predominant cause, with incidence increasing with age [1,3].

Clinically, AUR most often presents with suprapubic discomfort and inability to void, though symptoms may be nonspecific. Bedside bladder ultrasound provides a rapid, noninvasive method for confirming urinary retention and is widely used in the emergency department (ED). However, its accuracy may be limited in patients with obesity, ascites, or prior abdominal surgery. In such cases, cross-sectional imaging can provide additional diagnostic clarity and identify underlying structural contributors such as malignancy, fibrosis, or urethral obstruction [2,3].

Management of AUR centers on two priorities: immediate bladder decompression and identification of the underlying cause. Urethral catheterization is typically first-line; however, when this approach is unsuccessful or contraindicated, suprapubic catheterization serves as an effective alternative [5,6]. The percutaneous Seldinger technique is commonly employed and can be performed with ultrasound guidance, improving procedural safety and success, particularly in anatomically complex patients [4-6].

In patients with altered anatomy, such as prior abdominal surgery, mesh placement, or dense adhesions, standard suprapubic catheter placement may be technically challenging or unsuccessful. This case highlights the utility of adapting existing wire-guided access techniques and available equipment to overcome these challenges. The use of a small-diameter pigtail catheter, placed via the Seldinger technique under ultrasound guidance, provided effective and timely bladder decompression in a situation where both urethral and standard suprapubic approaches had failed.

Pigtail catheters are widely available in EDs, often included in chest tube or drainage kits, and are designed for wire-guided insertion. Their compatibility with standard guidewires and smaller caliber makes them particularly advantageous in patients where tissue resistance, fibrosis, or mesh may impede placement of larger suprapubic catheters [7,8]. This approach leverages procedural familiarity among emergency physicians, as the underlying technique mirrors central venous access and other commonly performed wire-guided procedures. Pigtail catheters are also well-described in other clinical applications, such as pleural drainage, where their smaller caliber and flexibility allow effective drainage with less invasiveness [9]. In this context, the present case extends these principles by demonstrating the successful use of a small-caliber pigtail catheter for bladder decompression in a patient with prior abdominal mesh and complex urologic anatomy. Compared to standard suprapubic catheterization, this approach offers a practical alternative when conventional techniques are not feasible, while maintaining familiarity with widely used procedural methods.

From an operational standpoint, this technique offers several advantages. It requires minimal additional training, utilizes readily available equipment, and allows for rapid bedside implementation. These factors are particularly relevant in emergency settings, where timely intervention is critical and access to specialized urologic equipment may be limited. Additionally, early decompression can significantly reduce patient discomfort and mitigate the risk of complications associated with prolonged urinary retention.

However, important limitations must be considered. The smaller diameter of pigtail catheters may limit their ability to drain blood clots effectively, posing a risk of clot retention in patients with hematuria or underlying malignancy. Furthermore, while the procedural risks are likely similar to those of standard percutaneous suprapubic catheterization, including infection, bleeding, and inadvertent injury, data specific to this technique in urinary applications remain limited.

Accordingly, the use of a pigtail catheter in this context should be considered a pragmatic, temporizing solution in select patients with difficult anatomy, rather than a definitive replacement for standard suprapubic catheterization. Further study is warranted to better define patient selection, safety profile, and long-term outcomes associated with this approach.

## Conclusions

In patients with AUR and complex anatomy, standard urethral and suprapubic catheterization may be unsuccessful. This case demonstrates that a small-caliber pigtail catheter, placed via an ultrasound-guided Seldinger technique, can provide an effective and readily available alternative for bladder decompression. This approach leverages familiar wire-guided techniques and may serve as a practical, temporizing solution in challenging clinical scenarios.

## References

[1] Dougherty JM, Leslie SW, Aeddula NR (2025). Male Urinary Retention: Acute and Chronic. https://www.ncbi.nlm.nih.gov/books/NBK538499/.

[2] Leissner J, Fisch M, Hohenfellner R (2006). Colonic pouch (Mainz-pouch III) for continent urinary diversion. BJU Int.

[3] Serlin DC, Heidelbaugh JJ, Stoffel JT (2018). Urinary retention in adults: evaluation and initial management. Am Fam Physician.

[4] Medical Advisory Secretariat (2006). Portable bladder ultrasound: an evidence-based analysis. Ont Health Technol Assess Ser.

[5] Ghaffary C, Yohannes A, Villanueva C, Leslie SW (2013). A practical approach to difficult urinary catheterizations. Curr Urol Rep.

[6] English SF (2017). Update on voiding dysfunction managed with suprapubic catheterization. Transl Androl Urol.

[7] Wagner AA, Godley ML, Duffy PG, Ransley PG (2004). A novel, inexpensive, double lumen suprapubic catheter for urodynamics. J Urol.

[8] Ojha V, Raju SN, Deshpande A, Ganga KP, Kumar S (2023). Catheters in vascular interventional radiology: an illustrated review. Diagn Interv Radiol.

[9] Gammie JS, Banks MC, Fuhrman CR, Pham SM, Griffith BP, Keenan RJ, Luketich JD (1999). The pigtail catheter for pleural drainage: a less invasive alternative to tube thoracostomy. JSLS.

